# Protective Effect of Eckol against Acute Hepatic Injury Induced by Carbon Tetrachloride in Mice

**DOI:** 10.3390/md16090300

**Published:** 2018-08-27

**Authors:** Shulan Li, Juan Liu, Mengya Zhang, Yuan Chen, Tianxing Zhu, Jun Wang

**Affiliations:** Department of Pharmacology, College of Medicine, Wuhan University of Science and Technology, Wuhan 430065, China; lishulan971204@outlook.com (S.L.); liujuan79@wust.edu.cn (J.L.); zhangmengya0703@163.com (M.Z.); susiechen1997@163.com (Y.C.); Yaoxue.xi@163.com (T.Z.)

**Keywords:** eckol, acute liver injury, apoptosis, oxidative stress, inflammation, dendritic cells

## Abstract

Several in vitro studies have shown the potential hepatoprotective properties of eckol, a natural phlorotannin derived from the brown alga. However, the in vivo hepatoprotective potential of eckol has not been determined. In this study, we performed an in vivo study to investigate the protective effect of eckol and its possible mechanisms on the carbon tetrachloride (CCl_4_)-induced acute liver injury model in mice. Results revealed that eckol pre-treatment at the dose of 0.5 and 1.0 mg/kg/day for 7 days significantly suppressed the CCl_4_-induced increases of alanine transaminase (ALT) and aspartate aminotransferase (AST) levels in serum and meliorated morphological liver injury. Terminal deoxynucleotidyl transferase-mediated dUTP nick end labelling (TUNEL) analysis showed that the number of positive apoptotic hepatocytes in the eckol-treated group was lower than that in the CCl_4_ model group. Western blotting analysis also demonstrated the enhanced expression of bcl-2 and suppressed expression of cleaved caspase-3 by eckol. The CCl_4_-induced oxidative stress in liver was significantly ameliorated by eckol, which was characterized by reduced malondialdehyde (MDA) formations, and enhanced superoxide dismutase (SOD), glutathione peroxidase (GSH-Px) activities and glutathione (GSH) content. Moreover, the CCl_4_-induced elevations of pro-inflammatory cytokines tumor necrosis factor (TNF)-α, interleukin (IL)-1β and IL-6 were markedly suppressed in the eckol-treated group. However, eckol enhanced the level of IL-10, a potent anti-inflammatory cytokine, and recruited CD11c^+^ dendritic cells into the liver tissues of CCl_4_-treated mice. These results indicated that eckol has the protective effect on CCl_4_-induced acute liver injury via multiple mechanisms including anti-apoptosis, anti-oxidation, anti-inflammation and immune regulation.

## 1. Introduction

**Figure 1:**
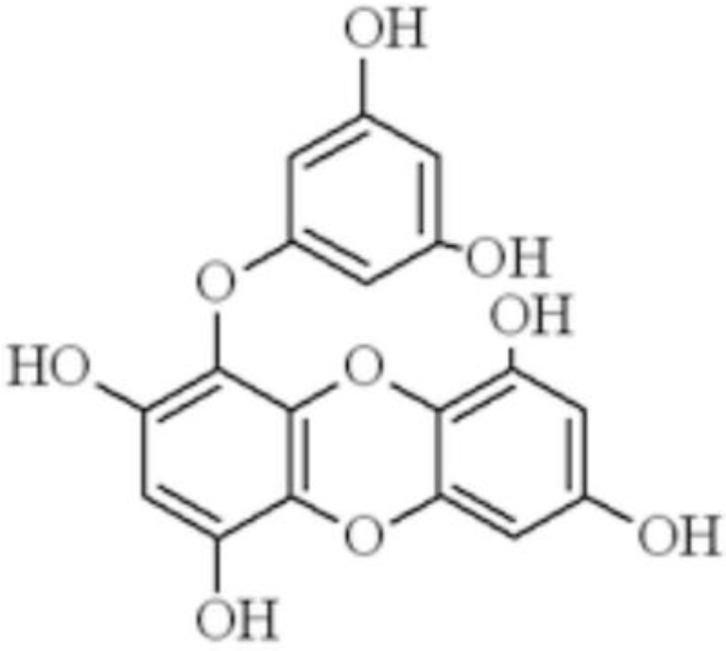
Structures of eckol.

As a trimer of phloroglucinol derived from the brown alga (e.g., *Ecklonia stolonifera*, *Eisenia bicyclis*, *Ecklonia cava*) [1,2,3,4,5], eckol (Figure 1) has been demonstrated to possess some pharmacological activities, such as anti-oxidative [2], anti-inflammatory [3,4], antithrombotic and profibrinolytic [5], radioprotective [6] properties, etc. In particular, several in vitro studies [7,8,9,10] have shown the potential hepatoprotective properties of eckol. In fact, as a main source of eckol, *Ecklonia stolonifera* has long been reported to be the only one with promising hepatoprotective activity among 18 studied seaweed variants [11]. Then, eckol was identified as one of principal constituents of *Ecklonia stolonifera* with hepatoprotective and antioxidant activities on tacrine-induced hepatotoxicity [7]. *Ecklonia stolonifera*-derived eckol has been found to exhibit protective effects on doxorubicin-induced hepatotoxicity in primary rat hepatocytes with EC_50_ values of 8.3 μg/mL, which were comparable to the well-known hepatoprotective agent silymarin (EC_50_ = 8.0 μg/mL), a polyphenolic flavonoid from milk thistle [8]. The antioxidant activity of eckol via suppressing the production of intracellular reactive oxygen species and enhancing glutathione levels contributed to its hepatoprotective effect in human hepatic HepG2 cells, a cell line which maintained most specialized functions like normal hepatocytes [9,10]. Kang et al. [4] investigated the anti-inflammatory activity of phlorotannins from *Eisenia bicyclis* on HepG2 cells stimulated by lipopolysaccharide (LPS). Among the four studied phlorotannins, only eckol was found to exhibit significant inhibition of LPS-induced inflammatory responses without any cytotoxicity, as indicated by decreased production of pro-inflammatory cytokines including interleukin (IL)-1, IL-6, tumor necrosis factor (TNF)-α and downregulated expression of inflammatory molecules such as cyclooxygenase (COX)-2 and inducible nitric oxide synthase (iNOS). Moreover, the ability of eckol to safeguard mitochondrial function in oxidative stress-damaged hepatocytes has been demonstrated in Chang liver cells [12]. Together, these previous in vitro data suggested eckol might be used as a novel natural therapeutic or preventive hepatoprotective agent. However, the in vivo hepatoprotective potential of eckol has not been determined.

Acute liver injury occurring within a short period is a common pathology of various liver diseases caused by toxins, drugs, alcohol, and pathogen infection, etc. Several cellular and molecular cascades of events, including oxidative stress, inflammation, hepatocyte apoptosis and necrosis, immune responses, etc. have been considered to contribute to the pathogenesis and progression of this disorder [13,14]. It is well-known that carbon tetrachloride (CCl_4_)-induced acute liver injury model shares a similar molecular mechanism with acute chemical liver injury in humans [15]. Especially, oxidative stress, inflammation response, and apoptosis have been recognized as the most important pathomechanisms during the progress of CCl_4_-induced acute liver injury [16]. Within the liver, CCl_4_ as a hepatotoxicant is metabolized by the cytochrome P450 enzyme to a highly reactive trichloromethyl free radical (•CCl_3_) and/or trichloromethyl peroxyl radical (•OOCCl_3_), which sequentially attacks hepatic tissue, leading to liver lipid peroxidation and oxidative damage [16]. In addition, CCl_4_ exposure could trigger the production of inflammatory cytokines and chemokines, stimulating the recruitment of inflammatory cells [16]. Moreover, as another crucial event involved in the acute liver injury induced by CCl_4_, apoptosis is a programmed cell death leading to morphological changes and death in the hepatocytes [17]. Accordingly, antioxidant, anti-inflammatory and anti-apoptosis therapies have been shown to have desirable effects for prevention or treatment of liver diseases in animal models, and to be clinically effective in preventing the disease progress or improving the outcome of patients [18,19]. Therefore, in view of the uncovered anti-oxidant [9,10,12] and anti-inflammatory [3] activities of eckol in hepatocytes, in this study, the CCl_4_-induced murine model was chosen to investigate the in vivo protective effect of eckol and its possible mechanisms on acute liver injury.

## 2. Results and Discussion

### 2.1. Eckol Ameliorates Acute Liver Injury Induced by CCl_4_ Exposure

The hepatoprotective effects of eckol pre-treatment (at the dose of 0.5 and 1.0 mg/kg/day for 7 days) on CCl_4_-induced acute liver injury were firstly assessed using serum levels of alanine transaminase (ALT) and aspartate aminotransferase (AST), together with the liver histology. As the commonly-used biochemical markers of liver injury in the clinics, the serum ALT and AST levels are elevated when the permeability of the hepatocellular membrane increases and the structural integrity of hepatocytes is damaged [20]. As shown in Table 1, CCl_4_ exposure dramatically enhanced the serum activities of both transferases (*p* < 0.01 vs. normal control group). However, these CCl_4_-induced increases of ALT and AST levels in serum were significantly blocked in low-, high-dose eckol and bifendate treated mice (*p* < 0.05 or *p* < 0.01, vs. CCl_4_ model group), suggesting that eckol has protective activity against CCl_4_-induced impairment of liver function. The effects of eckol (1.0 mg/kg) were comparable to bifendate (200 mg/kg), a positive control which is a synthetic intermediate of schisandrin C with antioxidant, anti-apoptosis and anti-inflammatory properties, which has been demonstrated to protect against liver injury induced by drugs and CCl_4_ in animals, and is widely used for the treatment of hepatitis and other liver diseases in clinical practice [21].

**Figure 2:**
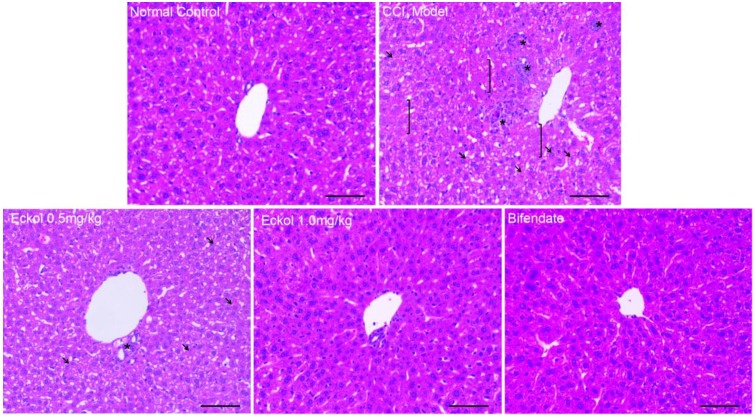
Effects of eckol on CCl_4_-induced liver histopathological changes. Representative hematoxylin-eosin (HE)-stained sections of liver sections. Symbols: brackets (]): areas with necrosis; black arrow: tumefaction and microvesicular steatosis; asterisks (*): inflammatory infiltration. Scale bar = 100 μm.

Histological analysis showed that CCl_4_ exposure induced severe damage in the liver parenchyma, which was manifested as scattered areas of necrosis, obvious hepatocyte edema, loss of cellular boundaries and destruction of hepatic architecture (Figure 2). Bifendate could significantly relieve the liver injury, and the hepatocyte structure was basically normal in the bifendate-treated group. Compared with the CCl_4_ model group, the pre-treatment of eckol meliorated morphological liver injury, as indicated by milder necrosis and well-preserved hepatic structure. Likewise, as shown in Table 2, pre-treatment of low-, high-dose eckol, or bifendate, resulted in normalization of hepatic architecture and significant reductions in the histological scores (*p* < 0.01, vs. CCl_4_ model group). 

### 2.2. Eckol Protects Hepatocytes from CCl_4_-Mediated Apoptosis

**Figure 3:**
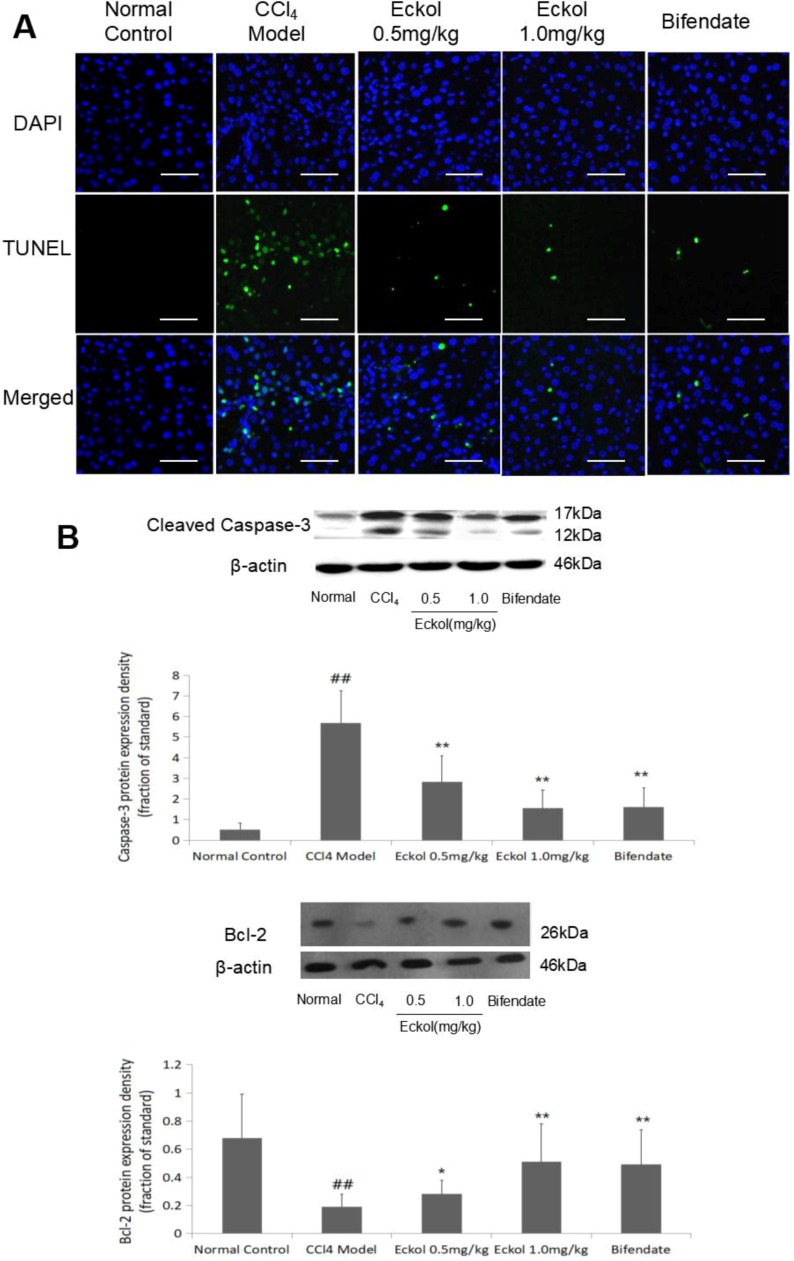
Effects of eckol on CCl_4_-induced hepatocyte apoptosis (**A**,**B**). (**A**) TUNEL analysis was used to detect cell apoptosis. Scale bar = 100 μm. (**B**) The protein expressions of cleaved caspase-3 and bcl-2 were detected by western blotting. Data were represented as mean ± SD (*n* = 10). An analysis of the variance using the one way analysis of variance (ANOVA) followed by least significant difference (LSD) test was conducted to examine the difference. ## *p* < 0.01 vs. normal control group; * *p* < 0.05, ** *p* < 0.01 vs. CCl_4_ model group.

As the first cellular response to liver toxic damage, hepatocyte apoptosis is one of the key contributing factors for the development of acute liver injury and is also a significant feature in CCl_4_-induced liver injury [17,22,23]. The results from terminal deoxynucleotidyl transferase-mediated dUTP nick end labelling (TUNEL) assay confirmed that CCl_4_ challenge induced massive hepatocyte apoptosis; importantly, pre-treatment with low-, high-dose eckol, or bifendate greatly reduced the number of TUNEL-positive apoptotic hepatocytes (Figure 3A).

To confirm the protective effect of eckol on hepatocyte apoptosis induced by CCl_4_ challenge, we further detected the expression levels of two key apoptosis-associated molecules (caspase-3 and bcl-2) in liver tissue by Western blotting (Figure 3B). In line with the results from the TUNEL assay, the significantly upregulated protein level of pro-apoptotic cleaved caspase-3 following CCl_4_ challenge was obviously decreased, while the downregulated protein level of anti-apoptosis gene bcl-2 following CCl_4_ challenge was increased by bifendate (*p* < 0.01, vs. CCl_4_ model group) and eckol at the dose of 0.5 and 1.0 mg/kg (*p* < 0.05 or *p* < 0.01, vs. CCl_4_ model group).

The anti-apoptosis activity of eckol has been reported in gamma-ray radiation-induced V79-4 lung fibroblast cells [24], and in mouse intestinal cells damaged by a single whole-body irradiation in vivo [25,26]. Here, we also found the protective effect of eckol on hepatocyte apoptosis induced by CCl_4_. Considering the important role of hepatocyte apoptosis in CCl_4_-induced liver injury [17,22,23], it is reasonable to assume that eckol could function as an anti-apoptotic agent with a subsequent hepato-protective effect.

### 2.3. Eckol Relieves CCl_4_-Induced Hepatic Oxidative Stress

As the central organ of metabolism and detoxification, the liver is more vulnerable to oxidative stress caused by metabolites and toxins in the body [27]. Sustained oxidative stress provokes the alteration of biological molecules including lipid, DNA and proteins, modulates biological pathways of gene transcription, protein expression and hepatocyte apoptosis, often finally leading to oxidative damage of the liver, therefore, has been regarded as a crucial factor in the initiation and development of hepatic disorder, regardless of the etiology [27,28]. As the imbalance between productions of reactive oxygen species or reactive nitrogen species and their elimination by antioxidant defense, oxidative stress could be evaluated by some indices, including malondiadehyde (MDA) (a reactive intermediate generated from lipid peroxidation), glutathione (GSH) (a non-enzymatic antioxidant), superoxide dismutase (SOD) and glutathione peroxidase (GSH-Px) (two antioxidant enzymes). 

Polyphenols mainly derived from plants are known natural compounds with potent antioxidant activity [2,28]. As electron-rich compounds, polyphenols are prone to enter into efficient electron-donation reactions in the presence of oxidizing agents, producing phenoxyl radical species as intermediates, which are further stabilized by resonance delocalization of unpaired electron to the *ortho* and *para* positions of the ring, or by hydrogen bonding with an adjacent hydroxyl group [2]. In this study, it is not surprising to find the attenuation of CCl_4_-induced hepatic oxidative stress by the pretreatment with eckol, which has a polyphenolic structure [2]. As shown in Table 3, CCl_4_ exposure significantly elevated the level of MDA, a commonly known marker of oxidative stress, depleted the endogenous antioxidant GSH, and suppressed the activities of endogenous antioxidants including SOD and GSH-Px in liver tissues (*p* < 0.01, vs. normal control group). Importantly, eckol pre-treatment at 0.5 and 1.0 mg/kg for 7 consecutive days dose-dependently ameliorated all of these alterations of hepatic oxidative stress induced by CCl_4_ (*p* < 0.05 or *p* < 0.01, vs. CCl_4_ model group). These data suggested that the anti-oxidative activity of eckol might, at least partly, be responsible for its hepatoprotective effect.

### 2.4. Eckol Regulates the Levels of Cytokines in Livers of CCl_4_-Treated Mice

As an essential component of immune response, inflammation is another crucial event associated with liver diseases [27]. One of key pathological characteristics of hepatic inflammation is the infiltration of immune/inflammatory cells into the liver, which is primarily responsible for fighting against pathogenic invasion and maintaining healthy tissue, however, in turn can lead to severe cellular injury when out of control [27]. The anti-inflammatory property of eckol has been reported in human HepG2 cells stimulated by LPS [3]. A recent study [4] also showed that eckol attenuated the expression of inflammatory cytokines such as TNF-α, IL-1β, IL-6 and IL-8 in human epidermal keratinocytes stimulated with the airborne particulate matter with a diameter of <10 μm.

**Figure 4:**
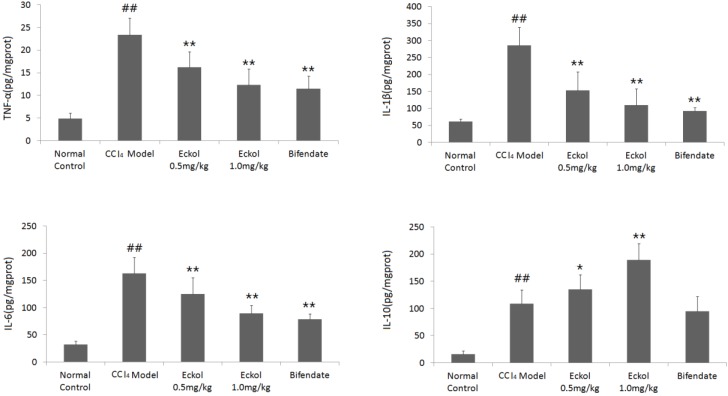
Effects of eckol on the levels of cytokines in livers of CCl_4_-treated mice. Data were represented as mean ± SD (*n* = 10). An analysis of the variance using the one-way analysis of variance (ANOVA) followed by least significant difference (LSD) test was conducted to examine the difference. ## *p* < 0.01 vs. normal control group; * *p* < 0.05, ** *p* < 0.01 vs. CCl_4_ model group.

Here, we studied the effect of eckol on the inflammation-related cytokines in the livers of CCl_4_-treated mice. As shown in Figure 4, the CCl_4_ model group exhibited significantly higher levels of cytokines (TNF-α, IL-1β, IL-6 and IL-10) in the liver (*p* < 0.01, vs. normal control group). Additionally, CCl_4_-induced elevations of pro-inflammatory cytokines TNF-α, IL-1β and IL-6 were markedly suppressed in the eckol-treated group (*p* < 0.01, vs. CCl_4_ model group). However, eckol further notably enhanced the level of IL-10 (*p* < 0.01, vs. CCl_4_ model group), a potent anti-inflammatory cytokine which plays a critical role in natural defense against detrimental immune/inflammatory responses [29], which was different from the positive control bifendate. 

### 2.5. Eckol Recruites Dendritic Cells into the Liver Tissues

The above finding, that eckol exhibited the activity of upregulating endogenous production of the anti-inflammatory cytokine IL-10, attracted our attention. In fact, exogenous administration of IL-10 has long been demonstrated to protect the liver from toxic damage caused by a number of insults [29]. It has been well-accepted that most of the inflammatory liver diseases are immune-mediated [30]. Importantly, one of the main sources of IL-10 production in liver is CD11c^+^ dendritic cells, which are potent antigen-presenting cells that play a key role in the regulation of immune and inflammatory responses [31]. Generally, dendritic cells localized in healthy livers display a predominant tolerogenic phenotype, which is characterized by a low ability to endocytose antigens and to stimulate T lymphocytes, together with the higher production of immune- inhibitory mediators such as IL-10 [30,32]. Coincidentally, a previous study [33] had reported the in vitro immunomodulatory effects of eckol on the dendritic cells, one of which was that eckol could protect dendritic cells against apoptosis in a concentration dependent manner. 

**Figure 5:**
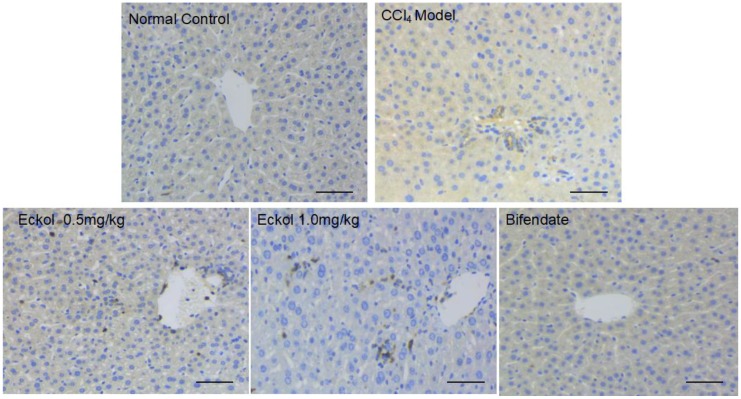
Effect of eckol on the immunohistochemical expression of CD11c, a commonly used and classical dendritic cell-specific marker, in livers of CCl_4_-treated mice. Scale bar = 100 μm.

In order to understand whether the protective effect of eckol against acute hepatic injury was related to dendritic cells, we performed the immunohistochemical staining for CD11c, a commonly used and classical dendritic cell-specific marker [34]. As shown in Figure 5, in the eckol-treated groups, more liver cells expressing CD11c were identified to infiltrate into the portal area and parenchyma of the liver, suggesting the dendritic cell recruitment activity of eckol might be responsible for the enhanced production of anti-inflammatory IL-10 in liver tissues, and therefore, might be a part of mechanisms underlying the protective effect of eckol against acute hepatic injury induced by CCl_4_.

## 3. Materials and Methods

### 3.1. Chemicals

CCl_4_ was purchased from Jiangsu Qiangsheng Chemical Co., Ltd (Jiangsu, China). Eckol was provided from Rongbao Environmental Technology Co., Ltd (Wuhan, China). Bifendate was obtained from Beijing Union Pharmaceutical Factory (Beijing, China).

### 3.2. Animal and Treatment

Male Kunming mice weighting 20 ± 2 g were obtained from the Laboratory Animal Center of Hubei Province (Wuhan, China). The study protocol was approved by the Institutional Animal Care and Use Committee at Wuhan University of Science and Technology (approval number 2018039). After an acclimation period of 1 week, mice were randomly divided into 5 groups (*n* = 10 in each group) as following: (1) normal control; (2) CCl_4_ model group; (3) CCl_4_ + eckol 0.5 mg/kg/day group; (4) CCl_4_ + eckol 1.0 mg/kg/day group; and (5) CCl_4_ + bifendate group. Before the CCl_4_ challenge, mice in 3 and 4 groups were orally pre-treated with eckol at the doses of 0.5 and 1.0 mg/kg/day, respectively, for 7 consecutive days. As the positive control, bifendate was dissolved in distilled water, and orally administrated to mice in 5 group at dose of 200 mg/kg/day for 7 days. In order to induce acute liver injury, mice in 2–5 groups were intraperitoneally injected with 0.5% CCl_4_ dissolved in olive oil at the dose of 10 mL/kg at 2 h after the last pretreatment administration. The mice in the normal control group were administrated with an equal volume of vehicle (intraperitoneally injected with olive oil + orally administrated with distilled water). At 24 h after CCl_4_ administration, all animals were sacrificed after collection of blood samples from the eyeballs. Liver samples were immediately harvested. 

### 3.3. Measurement of Serum AST and ALT

The collected blood samples were centrifuged at 3000 rpm for 15 min at 4 °C to separate the serums. The levels of serum ALT and AST were measured using enzymatic colorimetric methods according to their respective kit protocols (Nanjing Jiancheng Bio-Engineering Co., Ltd., Nanjing, China).

### 3.4. Histological Examination

Parts of each liver sample was kept in 10% neutral buffered formaldehyde solution for 24 h, then dehydrated in a graded series of alcohol and finally in xylene. Liver samples were further processed using the paraffin slice technique and the tissue was cut into 4–6 μm thickness. The sectioned tissue was stained with hematoxylin-eosin (HE) for histological examination of liver injury. The degree of liver injury was evaluated using a histological scoring system introduced by Corrêa-Ferreira et al [35]. This scoring system comprises three histological features: microvesicular steatosis grade (<5%, score 0; 5–33%, score 1; 33–66%, score 2; >66%, score 3); tumefaction grade (none, score 0; few, score 1; many, score 2); inflammatory grade (slight, score 0; moderate, score 1; marked, score 2; very marked, score 3).

### 3.5. Detection of Apoptotic Hepatocytes

The apoptotic cells in liver sections were detected using a commercial the terminal deoxynucleotidyl transferase mediated dUTP nick end labelling (TUNEL) staining kit according to the manufacturer’s instruction (Roche, Indianapolis, IN, USA). The sections were co-stained with the nuclei marker 4,6-diamidino-2-phenylindole (DAPI) (Servicebio, Wuhan, China).

### 3.6. Western Blotting

The liver tissues were homogenized, then centrifugated at 14,000 rpm for 20 min. The protein concentration in the supernatants was measured using the bicinchoninic acid assay kit (Nanjing Jiancheng Bio-Engineering Co., Ltd., Nanjing, China). The proteins were separated by 10% SDS-PAGE, then transferred to a nitrocellulose membrane by electroblotting. After blocking with TBS-T (20 mM Tris-HCl, 0.1% Tween 20, and 137 mM NaCl) containing 5% non-fat dry milk for 1 h at room temperature, the membrane was incubated with primary antibodies of cleaved caspase-3 (Cell Signaling Technology, Boston, MA, USA), bcl-2, and β-actin (Santa Cruz, Dallas, TX, USA) for 2 h, and then incubated with a secondary antibody horseradish peroxidase-conjugated anti-rabbit IgG/ anti-mouse IgG (Santa Cruz, Dallas, TX, USA) for 2 h at room temperature. Finally, the membrane was treated with the reagents in an electrogenerated chemiluminescence (ECL) chemiluminescence detection kit (Advansta, Menlo Park, CA, USA) then scanned. The relative amount of caspase-3 and bcl-2 was corrected with the amount of β-actin in the same sample. 

### 3.7. Measurement of Parameters Related to Oxidative Stress in the Liver

Partial hepatic tissues were homogenized on ice. The liver homogenates were subjected to measure the level of MDA and GSH, the activities of SOD and GSH-Px according to their respective manufacturer’s instructions (Nanjing Jiancheng Bio-Engineering Co., Ltd., Nanjing, China). The protein contents in liver homogenates were measured using the bicinchoninic acid assay kit (Nanjing Jiancheng Bio-Engineering Co., Ltd., Nanjing, China).

### 3.8. Measurement of TNF-a, IL-1β, IL-6 and IL-10 Levels

The levels of TNF-a, IL-1β, IL-6 and IL-10 in the liver homogenates were detected using ELISA kits according to the manufacturer’s instructions (R&D Systems, Minneapolis, MN, USA).

### 3.9. Immunohistochemistry

The paraffin liver sections (4 μm thick) were dewaxed, rehydrated, then followed by quenching of the endogenous peroxidase activity, blocking with 3% defatted dry milk, and incubating with the rabbit anti-CD11c antibody (Servicebio, Wuhan, China) at a dilution of 1:400 overnight at 4 °C. After washing, the slides were treated with the biotinylated goat anti-rabbit secondary antibody (Servicebio, Wuhan, China) for 2 h at room temperature. Immune complexes were visualized by incubation with 3,3′-diaminobenzidine tetrachloride (DAB). Then, sections were counter-stained with hematoxylin, dehydrated, coverslipped, and viewed under a microscope. 

### 3.10. Statistical Analysis

All data were represented as mean ± SD. The results were subjected to an analysis of the variance using the one-way analysis of variance (ANOVA) followed by least significant difference (LSD) test to analyze the difference. *p* < 0.05 were considered statistically significant.

## 4. Conclusions

In summary, in the present study, we demonstrated that eckol, a marine phlorotannin, has the protective effect on CCl_4_-induced acute liver injury, which was indicated by the decreased levels of serum ALT and AST, as well as the improved liver histology resulting from eckol pretreatment. Moreover, the results of our study implicated that multiple mechanisms might be involved in the in vivo hepatoprotective effect of eckol: (1) anti-apoptosis; (2) anti-oxidation; (3) anti-inflammation; (4) the recruitment of CD11c^+^ cells. The first three mechanisms are in conformity with previously published in vitro data on the biological activities of eckol [2,3,4,9,10,12,24], and are comparable to those observed in the bifendate group. However, the last demonstrates for the first time that the potential immunomodulatory roles of eckol in recruiting dendritic cells into the liver tissues and thus promoting anti-inflammatory IL-10 production, which were not observed in bifendate group, might also underlie the protective effect of eckol against acute liver injury induced by CCl_4_.

## Figures and Tables

**Table 1:** Effect of eckol on the serum levels of transaminases ALT and AST in CCl_4_-treated mice (*n* = 10, mean ± SD).

Groups	Doses	ALT(U/L)	AST(U/L)
Normal control	-	9.86 ± 2.74	18.65 ± 7.21
CCl_4_ model	-	129.37 ± 38.91 ##	117.22 ± 15.78 ##
Eckol	0.5 mg/kg	75.58 ± 23.45 **	86.79 ± 26.38 *
	1.0 mg/kg	49.43 ± 16.03 **	63.21 ± 18.89 **
Bifendate	200 mg/kg	50.34 ± 18.35 **	61.05 ± 28.61 **

## *p* < 0.01 vs. normal control group; * *p* < 0.05, ** *p* < 0.01 vs. CCl_4_ model group.

**Table 2:** Effect of eckol on the histological alterations in CCl_4_-treated mice (*n* = 10, mean ± SD).

Groups	Doses	Necrosis	Microvesicular Steatosis	Tumefaction	Inflammatory Infiltration
Normal control	-	−	-	0	0
CCl_4_ model	-	+	1.7 ± 0.5	1.8 ± 0.4	1.9 ± 0.6
Eckol	0.5 mg/kg	−	1.0 ± 0.7 **	1.2 ± 0.6 **	1.0 ± 0.7 **
	1.0 mg/kg	−	0.8 ± 0.7 **	0.8 ± 0.7 **	0.6 ± 0.5 **
Bifendate	200 mg/kg	−	0.9 ± 0.8 **	0.8 ± 0.7 **	0.8 ± 0.7 **

* *p* < 0.05, ** *p* < 0.01 vs. CCl_4_ model group.

**Table 3:** Effect of eckol on the levels of hepatic MDA, GSH, SOD and GSH-Px in CCl_4_-treated mice. (*n* = 10, mean ± SD).

Groups	Doses	MDA (nmol/mg prot)	GSH (nmol/mg prot)	SOD (U/mg prot)	GSH-Px (U/mg prot)
Normal control	-	2.46 ± 0.62	119.32 ± 34.35	143.69 ± 30.81	101.49 ± 16.38
CCl_4_ model	-	4.55 ± 0.73 ##	61.38 ± 29.91 ##	91.52 ± 26.37 ##	50.26 ± 13.87 ##
Eckol	0.5 mg/kg	3.62 ± 1.09 *	88.59 ± 28.32 *	112.36 ± 37.72	76.56 ± 20.97 **
	1.0 mg/kg	2.86 ± 0.74 **	93.78 ± 25.50 *	139.79 ± 45.52 **	92.67 ± 29.38 **
Bifendate	200 mg/kg	3.73 ± 0.96 *	85.39 ± 26.69	106.89 ± 38.21	74.55 ± 32.73 *

## *p* < 0.01 vs. normal control group; * *p* < 0.05, ** *p* < 0.01 vs. CCl_4_ model group.
